# Protein oxidation, glycation, and carbamylation in patients with adrenal masses

**DOI:** 10.3389/fmolb.2026.1811270

**Published:** 2026-07-09

**Authors:** Barbara Choromańska, Piotr Myśliwiec, Marta Lewoc, Jacek Dadan, Katarzyna Choromańska, Anna Zalewska, Mariola Matulewicz, Małgorzata Żendzian-Piotrowska, Mateusz Maciejczyk

**Affiliations:** 1 Department of General and Endocrine Surgery, Medical University of Bialystok, Bialystok, Poland; 2 Department of Oral Surgery, Medical University of Gdansk, Gdansk, Poland; 3 Experimental Dentistry Laboratory, Medical University of Bialystok, Bialystok, Poland; 4 Regional Centre for Transfusion Medicine in Bialystok, Bialystok, Poland; 5 Department of Hygiene, Epidemiology and Ergonomics, Medical University of Bialystok, Bialystok, Poland

**Keywords:** adrenal tumors, carbamylations, disulfide, glycoxidation, thiol

## Abstract

Adrenal masses are the most common of all tumors in the adult population, and their size is associated with malignancy. The knowledge about the pathogenesis of adrenal tumors is limited. Objectives: Evaluation of the association between protein glycooxidation and carbamylation, as well as thiol-disulfide homeostasis and adrenal tumor size. The study group included 71 patients (39 women and 32 men) with adrenal masses, divided into two groups: 34 patients with adrenal masses <4 cm in diameter and 37 patients with adrenal masses >4 cm in diameter. Colorimetric and spectrophotometric methods were used in this study. The protein oxidative damage (increase in ischemia-modified albumin) and glycooxidation rate (increase in kynurenine, dityrosine, Amadori products, advanced glycation end products, and carbamyl-lysine [CBL]) were significantly higher in patients with adrenal masses than in comparison with the control group. Additionally, thiol-disulfide homeostasis (decrease in total thiols and native thiols and increase in disulfide) was disturbed in these patients. The plasma content of CBL seemed to express possible diagnostic utility. This parameter, with 75.68% sensitivity and 74% specificity, differentiates patients with adrenal tumors (both AM < 4 cm and AM > 4 cm) from the controls. Moreover, plasma content of CBL has a diagnostic value in distinguishing AM < 4 cm from those AM > 4 cm. In conclusion, protein glycoxidation and thiol-disulfide homeostasis are impaired in patients with adrenal tumors. Although oxidative stress is involved in the progression of carcinogenesis, oxidation and glycoxidation of plasma proteins, as well as thiol-disulfide homeostasis, were not dependent on tumor size. Only the evaluation of CBL plasma content seems helpful in predicting the malignant risk of adrenal masses. More research is needed to confirm our results.

## Introduction

Adrenal tumors, due to their non-specific symptoms, are a serious health problem. Although most of them are benign and hormonally inactive, some may have hormonal activity, with overproduction of cortisol (or other steroids), aldosterone, or catecholamines (Grumbach et al., 2003; Jason and Oltmann, 2019; Ebbehoj et al., 2020). These hormones play an important role in many physiological processes. Cortisol is primarily anti-inflammatory. Under stress, it affects the metabolism of carbohydrates, lipids, and proteins, increasing the concentration of glucose and intensifying lipolysis of adipose tissue (Jonczyk, 2014; Bin Rubaia’an et al., 2021). Catecholamines act as neurotransmitters and take part in the regulation of blood circulation, whereas aldosterone is involved in the regulation of water and mineral balance (Erlic and Beuschlein, 2019; Karwacka et al., 2021). Excessive secretion of these hormones by adrenal tumors may be associated with severe morbidity and even mortality (Clayton, 2022). The risk of malignancy depends on the size of the adrenal mass. In the report from the National Italian Study Group, selecting a 4 cm cutoff had a sensitivity of 93% in detecting adrenocortical carcinoma (Angeli et al., 2008). In most cases, complete surgical resection is the only potentially effective treatment for adrenal masses.

Until now, little has been known about the pathogenesis of adrenal tumors. Risk factors for developing these tumors include hormonal contraceptives in women, smoking, and genetic abnormalities. The mutation of the VHL/HIF axis as a risk factor for the development of pheochromocytoma has been the most extensively studied (Vaidya et al., 2018). It has been shown that deregulation of the hypoxia-inducible factor (HIF-1) may have a different effects on tumor growth and metabolism, as well as on metastases (Fishbein et al., 2017). HIF-1α exerts different effects depending on the amount of oxygen delivered to cells (Movafagh et al., 2015). Under conditions of normal cellular oxygen levels, it is degraded by the ubiquitin-proteasome pathway. However, hypoxia leads to its excessive accumulation and, consequently, to the disruption of many genes involved in cancer pathogenesis (Rashid et al., 2021). In our previous studies, we found that the pathogenesis of adrenal masses may be associated with an antioxidant/oxidative imbalance in patients with different types of adrenal tumors (non-functional incidentaloma, pheochromocytoma, and Cushing’s/Conn’s adenoma) (Choromańska et al., 2021a; 2021b). Excessive production of reactive oxygen species (ROS) and reactive nitrogen species (RNS) is known to initiate the process of lipid and protein oxidation (Förstermann, 2008; Choromańska et al., 2020; Zińczuk et al., 2020). It is important to mention the detrimental effect of the glycooxidation process on cellular biomolecules. Glycooxidation exerts the most toxic effect on proteins, as it causes their aggregation, denaturation, and modification/loss of physiological function (Stadtman and Levine, 2003). Moreover, accumulation of highly cytotoxic products of protein glycooxidation leads to further cellular damage and consequently, to the dysregulation of multiple signaling pathways, initiation of inflammatory processes, and cell death (El-Bikai et al., 2010; Maciejczyk et al., 2020). The importance of the carbamylation process in the development of various diseases is also emphasized. Carbamylation is a post-translational modification of proteins resulting from a non-enzymatic reaction in which isocyanic acid binds to free amino groups of proteins. This process, similarly to glycooxidation, can lead to an abnormal structure and function of proteins. In contrast, thiol-disulfide homeostasis has a protective function against irreversible oxidation of protein cysteine residues. Thiol-disulfide homeostasis, which reflects the levels of thiols and disulfides, relies on the reversal of the thiol oxidation process in proteins (Erel and Erdoğan, 2020). Dysregulated thiol-disulfide homeostasis has been observed in many diseases such as coronary atherosclerosis, Alzheimer’s disease, type 2 diabetes, and lung cancer (Kundi et al., 2015; Dirican et al., 2016; Gumusyayla et al., 2016; Ergin et al., 2020). Until now, nothing has been known about protein glycooxidation and the carbamylation process, as well as thiol-disulfide homeostasis in patients with adrenal tumors. It is also unclear whether disorders in these processes are associated with the progression and growth of adrenal tumors. Tumor size is considered to be the best predictor of malignancy in adrenal masses (Barnett Jr et al., 2000). It was shown that 6% of adrenal tumors in the size range of 4.1–6 cm may become malignant, whereas tumors larger than 6 cm may be malignant in as many as 25% of cases (Bhoster et al., 1932). Therefore, the aim of our study was to evaluate protein glycooxidation and thiol-disulfide homeostasis in patients with adrenal masses depending on the tumor size.

## Materials and methods

The study protocol conformed to the Guidelines for Good Clinical Practice and the Declaration of Helsinki, and was approved by the Bioethics Committee of the Medical University of Bialystok (permission code: R-I-002/66/2015, APK.002.341.2020). All patients provided informed consent to participate in this study.

The study group included 71 patients (39 women and 32 men aged 42–76 years) with adrenal masses, who had been diagnosed at internal medicine departments with an endocrinology profile. Preoperative workup included imaging with adrenal CT protocol, assessment of the daily cortisol rhythm, the dexamethasone suppression test, serum dehydroepiandrosterone, aldosterone and the aldosterone/renin ratio, electrolytes, as well as metanephrine and normetanephrine in daily urine. Patients underwent elective endoscopic adrenalectomy (via the lateral transperitoneal approach or the posterior retroperitoneal approach) at the First Department of General and Endocrine Surgery at the University Hospital in Bialystok, Poland. Patients were divided into two groups depending on the tumor size: 34 patients with adrenal masses <4 cm in diameter (AM < 4 cm) and 37 patients with adrenal masses >4 cm in diameter (AM > 4 cm). The cutoff value of 4 cm was used to divide the patients with adrenal tumors into two groups. This threshold was based on studies suggesting that adrenal incidentalomas larger than 4 cm should be considered for adrenalectomy because of the increased risk of malignancy (Mantero et al., 2000). These recommendations are consistent with the current Polish guidelines (Handkiewicz-Junak et al., 2024). Additional radiological features associated with a higher risk of malignancy include high unenhanced computed tomography attenuation values (>10 Hounsfield units), contrast washout below 60%, irregular shape, heterogeneous density, tumor calcifications, and rapid growth in consecutive imaging studies. Therefore, some adrenal masses smaller than 4 cm in our cohort were also classified as incidentalomas.

In the AM < 4 cm subgroup, 17 patients were diagnosed with incidentaloma, 5 with Cushing’s syndrome, 4 with Conn’s syndrome, and 8 with pheochromocytoma, whereas in the AM > 4 cm subgroup, 27 patients had incidentaloma, 5 had pheochromocytoma, 3 had Cushing’s syndrome and 2 had Conn’s syndrome. Patients suffering from Conn’s syndrome were treated with an aldosterone receptor blocker (spironolactone) and/or potassium supplementation in the preoperative period. Patients with pheochromocytoma received doxazosin (a selective alpha-1-adrenergic receptor blocker) for at least 14 days before surgery to prevent an intraoperative hypertensive crisis. The patients in the study group had creatinine results within the reference range.

Fifty healthy volunteers (25 women and 25 men aged 44–68) were assigned to the control group. They visited the Specialist Dental Clinic at the Medical University of Bialystok for routine dental follow-up examinations. These patients did not have periodontal disease or active caries. The full blood counts, INR, and biochemical blood parameters (creatinine, ALT, AST, K^+^, and Na^+^) were within the reference range.

The exclusion criteria for the control and study groups were: pregnancy among women, alcohol abuse, smoking, acute inflammation, infectious diseases (HIV/AIDS, hepatitis A, B and C) neoplastic diseases, cardiovascular diseases (other than hypertension), diseases of the respiratory, digestive and genitourinary systems, autoimmune diseases (ulcerative colitis, Crohn’s disease and Hashimoto’s disease) and metabolic diseases (gout, insulin resistance, type 1 diabetes, osteoporosis and mucopolysaccharidosis) and kidney diseases as well as intense physical activity within 24 hours before blood sampling. Patients in the study group had creatinine results within the reference range. Additionally, during the 3 months prior to sampling, all qualified patients did not take antioxidant supplements (including iron preparations), non-steroidal anti-inflammatory drugs, glucocorticosteroids, or antibiotics.

### Blood collection

All blood samples from healthy controls and patients with adrenal masses were collected in a fasting state into serum and EDTA pre-coated tubes (SARSTEDT, S-Monovette) and centrifuged at 4,000 rpm for 10 min at 4 °C to obtain serum and plasma. A measure of 10 μL of 0.5 M butylated hydroxytoluene (BHT) was added to 1 mL of plasma to avoid oxidation of the samples, which were then stored at −80 °C until final assessments (Zińczuk et al., 2020). ischemia-modified albumin (IMA) was determined in the serum, and the remaining parameters were assessed in plasma.

### Laboratory measurements

A Sysmex XN1000 automated blood analyzer was used to measure the full blood count. Biochemical tests (Na^+^, K^+^, cortisol before 10 a.m., serum aldosterone, urine metanephrine and normethanephrine, and glucose) were analyzed using an Abbott analyzer (Abbot Diagnostics, Wiesbaden, Germany). The laboratory tests of the controls and study group patients were assessed in the Laboratory of Biochemical Diagnostics, University Hospital in Bialystok, Poland. The clinical and laboratory characteristics of the controls and patients with adrenal masses are shown in Table 1.

**TABLE 1:** Laboratory characteristics of the controls, patients with adrenal masses <4 cm in diameter (AM < 4 cm), and patients with adrenal masses >4 cm in diameter (AM > 4 cm).

	Controls *(n = 50)*	*AM < 4 cm (n = 34)*	AM > 4 cm *(n = 37)*	ANOVA
Size of the tumor (cm)	-	2.6 ± 0.89	5.2 ± 1.1^^^^	*p < 0.0001*
Aldosterone (ng/dL)	11.75 ± 5.35	20 ± 11.51**	15.64 ± 8.97	*p = 0.0031*
Serum cortisol before 10 a.m. (µg/dL)	11.43 ± 4.19	15.88 ± 5.21***	13.76 ± 5.89	*p = 0.0008*
Urine methanephrine (µg/24h)	115.8 ± 64.67	254.4 ± 241.5**	212.3 ± 445.3	*p = 0.0044*
Urine normethanephrine (µg/24h)	232 ± 79.67	435.8 ± 305.9***	353.8 ± 224.5**	*p = 0.0002*
Glucose (mg/dL)	77.46 ± 5	95.9 ± 14.72****	107.3 ± 53.28****	*p < 0.0001*
Na^+^ (mmol/L)	139.9 ± 2.74	139.5 ± 2.45	139.6 ± 2.85	*p = 0.6395*
K^+^ (mmol/L)	4.35 ± 0.45	4.32 ± 0.49	4.462 ± 0.41	*p = 0.5266*
WBC (10^3^/μL)	6.75 ± 1.1	7.39 ± 1.91	7.45 ± 2.4	*p = 0.3043*
RBC (10^6^/μL)	4.534 ± 0.38	4.6 ± 0.39	4.56 ± 0.53	*p = 0.682*
HGB (g/dL)	13.87 ± 0.85	13.89 ± 1.22	13.94 ± 1.47	*p = 0.9007*
PLT (10^3^/μL)	288.6 ± 10.66	216.5 ± 55.42****	240.1 ± 61.15****	*p < 0.0001*
BMI (kg/m^2^)	23.81 ± 1.44	29.73 ± 5.64****	29.8 ± 6.28****	*p < 0.0001*

Results are presented as mean with standard deviation. **p < 0.01, ***p < 0.001 ****p < 0.0001 indicate significant differences from the controls. ^^^^ p < 0.0001 indicates significant differences from the (AM < 4 cm); white blood cell count (WBC), red blood cell count (RBC), hemoglobin (HGB), platelet count (PLT), and body mass index (BMI).

### Glycation products

The formation of amyloid cross-β structure and Amadori products was evaluated colorimetrically using a thioflavin T assay for amyloid (LeVine, 1999) and a nitro blue tetrazolium assay for Amadori products (Johnson and Baker, 1987). The characteristic fluorescence of amyloid was measured at 435/485 nm, and that of Amadori products at 525 nm, and an extinction coefficient of 12,640 cm^−1^ mol^−1^ L for monoformazan was used. The content of plasma advanced glycation end products (AGE) was measured spectrofluorimetrically at 350/440 nm by evaluating AGE-specific fluorescence (Kalousová et al., 2002).

### Glycoxidation products

Fluorescence evaluation of protein oxidative modifications was peformed. The tryptophan (TRY), dityrosine (DT), kynurenine (KYN), and N-formylkynurenine (NFKYN) contents were analyzed fluorimetrically. Immediately before measurement, blood plasma samples were diluted in 0.1 M H_2_SO_4_ (1:5, v/v). The characteristic fluorescence at 295/340 nm for TRY, 330/415 nm for DT, 365/480 nm for KYN, and 325/434 nm for NFKYN was measured in 96-well black-bottom microplates (Diplock and Rice-Evans, 1991). The results were expressed as arbitrary fluorescence units (AFU)/mg protein.

### Oxidation products

The content of protein carbonyls (PC) was assayed spectrophotometrically using 2,4-dinitrophenylhydrazine (2,4-DNPH) (Reznick and Packer, 1994). The absorbance of a stable complex of PC - 2,4-DNPH was measured at 593 nm. Serum IMA content was determined colorimetrically at 470 nm. The method uses the ability to bind exogenous cobalt (Co^2 +^) of human serum albumin (Bar-Or et al., 2000).

### Thiol/disulfide homeostasis

The content of total thiols (TT) and native thiols (NT) was simultaneously measured in a paired test. The concentration of native thiol groups was measured spectrophotometrically at 412 nm, using a modified Ellman’s reagent. In the parallel run, in the reaction with NaBH_4_, dynamic disulfide bonds were reduced to free thiol groups. The TT amount was measured after removing the unused reductant using formaldehyde. The amount of disulfide bonds (DS) was calculated as half the difference between the total amount of thiols and the NT (Erel and Neselioglu, 2014).

### Carbamylation products

The content of N^ε^-(carboxymethyl) lysine (CML) and carbamyl-lysine (CBL) was determined colorimetrically according to the manufacturer’s instructions using commercial enzyme-linked immunosorbent assay kits (Cell Biolabs, Inc. San Diego, United States).

### Statistical analysis

The sample size was determined *a priori* based on our previous pilot study. An online sample size calculator (ClinCalc) was used, and the minimum number of patients was 41 (level of significance = 0.05; power of study = 0.8).

The statistical package GraphPad Prism 9 for Mac (GraphPad Software, La Jolla, CA, United States) was used for statistical analysis. The distribution of the data was checked using the Shapiro–Wilk test, and the homogeneity of variance was assessed using Levene’s test. Due to the lack of normality in the data distribution, we used a non-parametric analysis of variance (ANOVA), namely, the Kruskal–Wallis test. The Dunn test was used for multiple comparisons, and the multiplicity-adjusted p-value was calculated. In the case of a normal distribution, ANOVA with Tukey’s *post hoc* was used. Pearson’s correlation coefficient was used to assess the correlation between the dependent variables. The assessment of the diagnostic utility of plasma biomarkers was based on receiver operating characteristic curves. The results for p < 0.05 were considered statistically significant.

## Results

Table 1 shows a comparison of the laboratory characteristics between the controls and patients with adrenal masses according to tumor size. Urine normethanephrine, serum glucose, and plasma PLT levels were significantly higher in patients with adrenal masses than in the controls. As there were more patients with hormonally active adrenal tumors in the AM < 4 cm group than in the AM > 4 cm group, we also observed increased serum aldosterone and cortisol concentrations, as well as urine methanephrine in patients with adrenal tumors in the AM < 4 cm compared with the controls. BMI was greater in patients with adrenal masses as compared with the controls.

**FIGURE 1:**
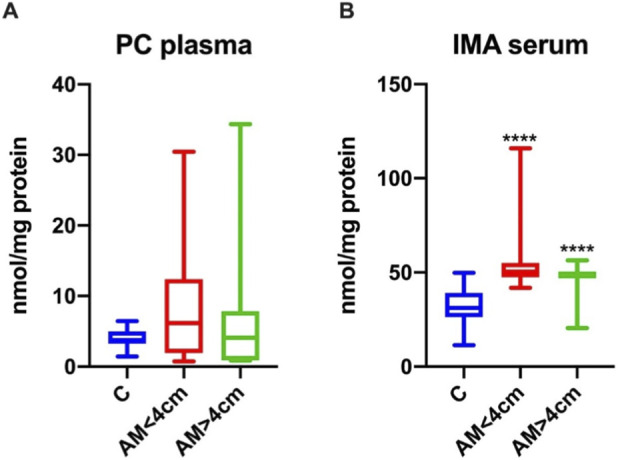
The plasma content of protein carbonyls (PC) **(A)** and serum content of ischemia-modified albumin (IMA) **(B)** in the controls **(C)** and patients with adrenal masses <4 cm in diameter (AM < 4 cm) and patients with adrenal masses >4 cm in diameter (AM > 4 cm). Results are presented as median with minimum and maximum. ****p < 0.0001 indicates a significant difference from the controls.

There were no differences in plasma PC content in any of the subgroups with adrenal masses compared to the control group (Figure 1A).

The content of serum IMA was significantly higher in patients with adrenal masses than in the controls (Figure 1B).

**FIGURE 2:**
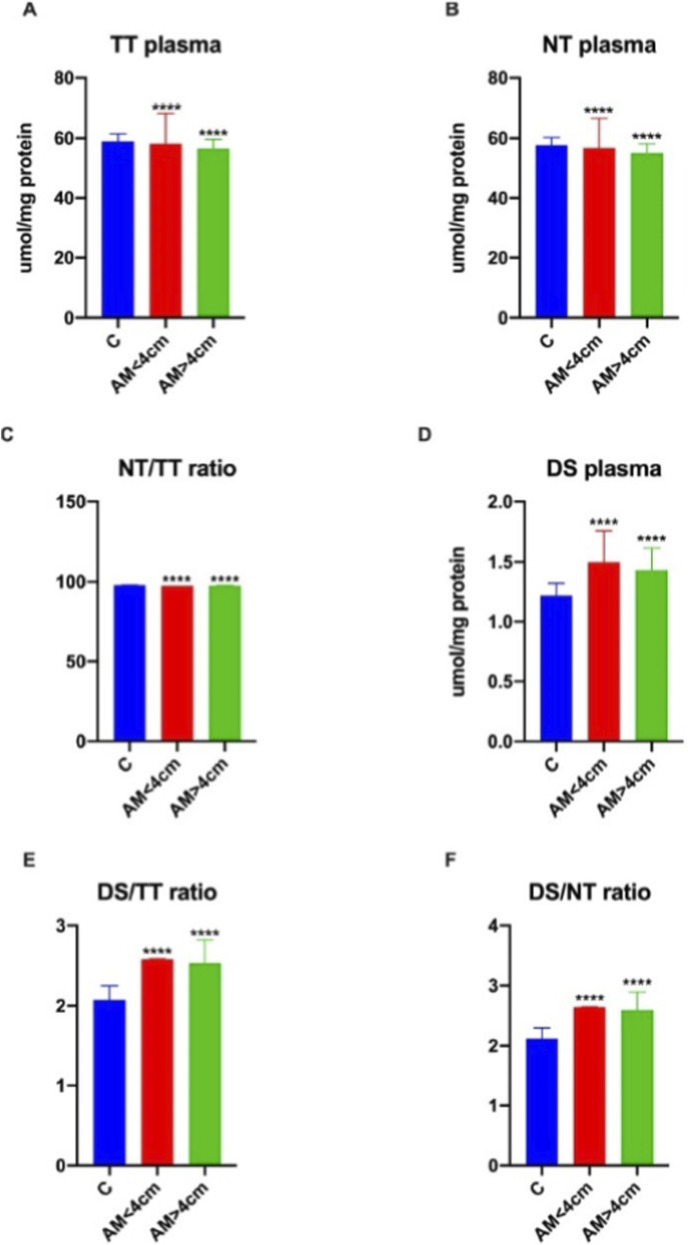
Plasma content of total thiols (TT) **(A)**, native thiols (NT) **(B)** and disulfide (DS) **(D)** as well as ratio NT/TT **(C)**, ratio DS/TT **(E)** and ratio DS/NT **(F)** in the controls (C) and patients with adrenal masses <4 cm in diameter (AM < 4 cm) and patients with adrenal masses >4 cm in diameter (AM > 4 cm). Results are presented as mean and standard deviation. ****p < 0.0001 indicates a significant difference from the controls. Patients with adrenal masses: AM < 4 cm (−25%, p < 0.0001) and AM > 4 cm (−27%, p < 0.0001) had lower content of plasma TRY than the controls (Figure 3A).

The plasma TT in patients with adrenal tumors AM < 4 cm (−2%, p < 0.0001) and AM > 4 cm (−5%, p < 0.0001) was decreased in comparison with the healthy controls (Figure 2A).

Similarly to TT, plasma content of NT was lower in patients with adrenal masses: AM < 4 cm (−2%, p < 0.0001) and AM > 4 cm (−5%, p < 0.0001) than the controls (Figure 2B).

The plasma DS content in adrenal masses subgroups: AM < 4 cm (+25%, p < 0.0001) and AM > 4 cm (+16%, p < 0.0001) was higher compared to the control group (Figure 2D).

Ratio of NT/TT plasma content was diminished in patients with adrenal masses: AM < 4 cm (−1%, p < 0.0001) and AM > 4 cm (−1%, p < 0.0001) (Figure 2C), while DS/TT and DS/NT plasma content ratio were markedly higher in subgroups with adrenal masses: AM < 4 cm (+24%, p < 0.0001; +24%, p < 0.0001, respectively) and AM > 4 cm (+19%, p < 0.0001; +24%, p < 0.0001, respectively) in comparison with the controls (Figures 2E,F).

**FIGURE 3:**
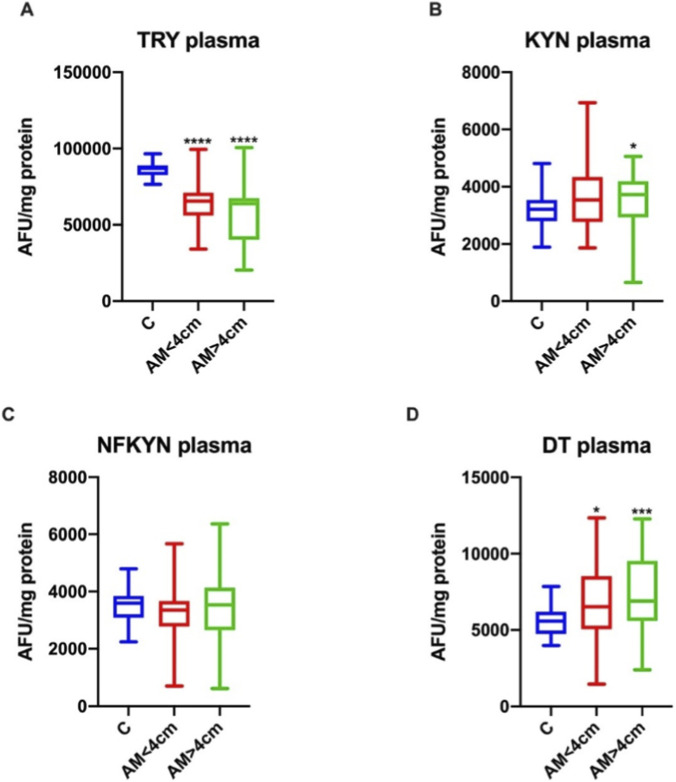
The plasma content of tryptophan (TRY) **(A)**, kynurenine (KYN) **(B)**, N-formylkynurenine (NFKYN) **(C)** and dityrosine (DT) **(D)** in the controls (C) and patients with adrenal masses <4 cm in diameter (AM < 4 cm) and patients with adrenal masses >4 cm in diameter (AM > 4 cm). Results are presented as median with minimum and maximum. *p < 0.05, ***p < 0.001, ****p < 0.0001 indicate significant differences from the controls.

Patients with adrenal mases: AM < 4 cm (-25%, p < 0.0001) and AM > 4 cm (-27%, p < 0.0001) had lower content of plasma TRY than the controls (Figure 3A).

The greater content of plasma KYN was only in patients with adrenal masses >4 cm in diameter (+16%, p = 0.0257) in comparison with the healthy controls (Figure 3B).

There were no statistically significant differences in the plasma content of NFKYN in groups of patients with adrenal masses as compared to the controls (Figure 3C).

In plasma of patients with adrenal tumors: AM < 4 cm (+17%, p = 0.0121) and AM > 4 cm (+24%, p = 0.0004), the content of DT was increased in comparison with the control group (Figure 3D).

**FIGURE 4:**
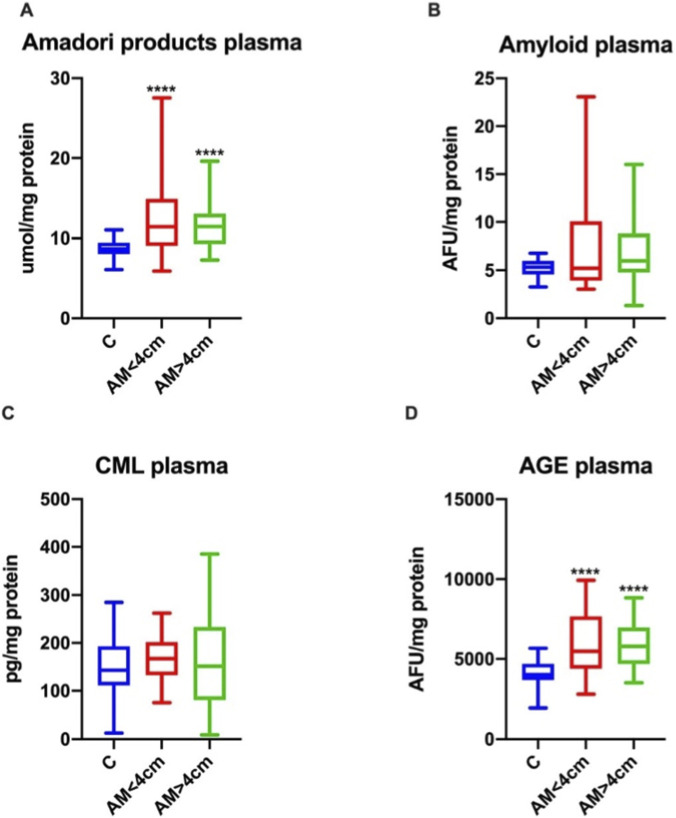
The plasma content of Amadori products **(A)**, amyloid **(B)**, N^ε^-(carboxymethyl) lysine (CML) **(C)** and advanced glycation end products (AGE) **(D)** in the controls (C) and patients with adrenal masses <4 cm in diameter (AM < 4 cm) and patients with adrenal masses >4 cm in diameter (AM > 4 cm). Results are presented as median with minimum and maximum. ****p < 0.0001 indicates significant difference from the controls.

Figure 4A shows greater plasma content of Amadori products in study groups: AM < 4 cm (+26%, p < 0.0001) and AM > 4 cm (+26%, p < 0.0001) than in the controls.

The plasma content of amyloid and CML in patients with adrenal masses did not differ from the control group (Figures 4B,C).

The plasma content of AGE was significantly increased in both subgroups of patients with adrenal masses: AM < 4 cm (+37%, p < 0.0001) and AM > 4 cm (+44%, p < 0.0001) as compared to the controls (Figure 4D).

**FIGURE 5:**
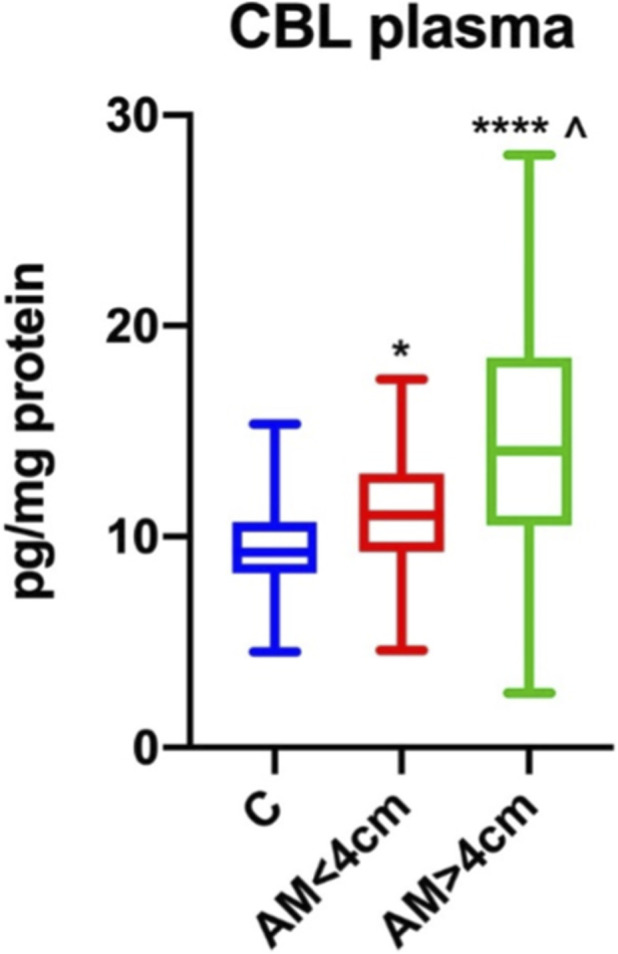
The plasma content of carbamyl-lysine (CBL) in the controls and patients with adrenal masses <4 cm in diameter (AM < 4 cm) and patients with adrenal masses >4 cm in diameter (AM>4 cm). Results are presented as median with minimum and maximum. *p < 0.05, ****p < 0.0001 indicate significant differences from the controls, ^ p < 0.05 indicates significant difference from the (AM < 4 cm).

The markedly higher plasma content of CBL was in patients with adrenal tumors: AM < 4 cm (+18%, p = 0.022) and AM > 4 cm (+51%, p < 0.0001) than the controls. Moreover, plasma content of CBL was higher in AM > 4 cm subgroup (+27%, p = 0.0347) in comparison with AM < 4 cm patients (Figure 5).

### Correlations

**FIGURE 6:**
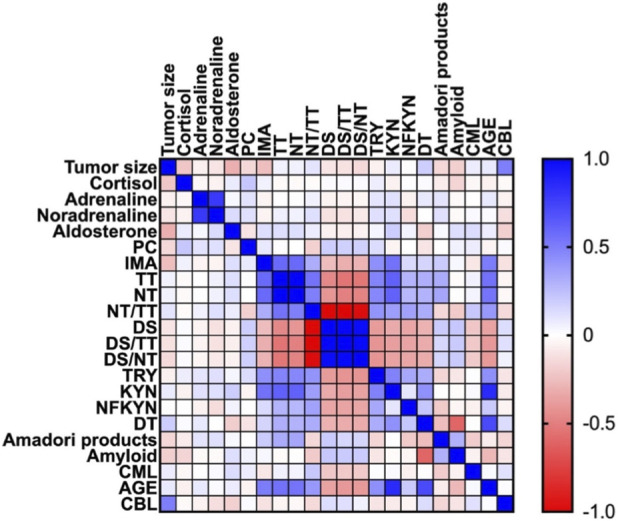
Correlations between the analyzed markers of protein glycation, glycoxidation, oxidation, and carbamylation products, as well as thiol-disulfide homeostasis; protein carbonyls (PC), ischemia-modified albumin (IMA), total thiols (TT), native thiols (NT), disulfide (DS), tryptophan (TRY), kynurenine (KYN), N-formylkynurenine (NFKYN), dityrosine (DT), N^ε^-(carboxymethyl) lysine (CML), advanced glycation end products (AGE), and carbamyl-lysine (CBL).

Correlations between the analyzed markers of protein glycation, glycoxidation, oxidation, and carbamylation products, as well as thiol-disulfide homeostasis, are shown in Figure 6.

In patients with adrenal masses, we found that tumor size positively correlated with plasma content of CBL (R = 0.496, p < 0.0001), and negatively with IMA (R = −0.252, p = 0.034) and plasma concentration of aldosterone (R = −0.313, p = 0.009).

### Glycation products

Amadori products were positively associated with amyloid (R = 0.298, p = 0.012), TT (R = 0.321, p = 0.006), and NT (R = 0.343, p = 0.003), and negatively with plasma content of DT (R = −0.255, p = 0.032). Negative correlation was also between plasma content of amyloid and DT (R = −0.603, p < 0.0001).

Plasma content of AGE was positively associated with plasma content of TT (R = 0.547, p < 0.0001), NT (R = 0.54, p < 0.0001), ratio of NT/TT (R = 0.399, p = 0.001), TRY (R = 0.435, p < 0.0001), KYN (R = 0.85, p < 0.0001) and DT (R = 0.71, p < 0.0001), DS (R = −0.361, p = 0.002), ratio of DS/TT (R = −0.4, p = 0.001) and DS/NT (R = −0.395, p = 0.001).

### Glycoxidation products

The positive correlations were between plasma content of TRY and TT (R = 0.477, p < 0.0001), NT (R = 0.467, p < 0.0001) and ratio of NT/TT (R = 0.414, p < 0.0001), as well as serum content of IMA (R = 0.434, p < 0.0001) and DT (R = 0.315, p = 0.008), whereas negatively with plasma content of DS (R = −0.386, p = 0.001), ratio of DS/TT (R = −0.416, p < 0.0001) and DS/NT (R = −0.415, p < 0.0001).

Plasma content of KYN and NYKYN correlated positively with plasma content of TT (R = 0.618, p < 0.0001; R = 0.291, p = 0.014), NT (R = 0.614, p < 0.0001, R = 0.276, p = 0.02), ratio of NT/TT (R = 0.376, p = 0.001; R = 0.358, p = 0.002), while negatively with DS (R = −0.328, p = 0.005; R = −0.345, p = 0.003), ratio of DS/TT (R = −0.374, p = 0.001; R = −0.356, p = 0.002) and DS/NT (R = −0.364, p = 0.002; R = −0.345, p = 0.003). Plasma content of KYN was also positively associated with plasma content of DT (R = 0.43, p < 0.0001).

### Oxidation products

Serum content of IMA was positively associated with plasma content of AEG (R = 0.507, p < 0.0001), KYN (R = 0.546, p < 0.0001), TT (R = 0.583, p < 0.0001), NT (R = 0.581, p < 0.0001) and ratio of NT/TT (R = 0.311, p = 0.008), while negatively with plasma content of DS (R = −0.261, p = 0.028), ratio of DS/TT (R = −0.306, p = 0.009) and DS/NT (R = −0.291, p = 0.014).

### Thiol/disulphide homeostasis

DT plasma content correlated positively with plasma content of TT (R = 0.307, p = 0.009), NT (R = 0.297, p = 0.012) and ratio of NT/TT (R = 0.315, p = 0.007), while negatively with plasma content of DS (R = −0.301, p = 0.011), ratio of DS/TT (R = −0.318, p = 0.007) and DS/NT (R = −0.321, p = 0.006).

In patients with adrenal masses, this study showed high positive correlations between TT and NT (R = 0.998, p < 0.0001). The plasma content of DS negatively correlated with TT (R = −0.462, p < 0.0001) and NT (R = −0.41, p < 0.0001).

### Clinical utility of CBL

Using multivariate regression analysis, we demonstrated that plasma content of CBL depended on tumor size. Results of multifactorial regression analysis are shown in Table 2.

**TABLE 2:** Multifactorial regression analysis of protein carbamylation rate in adrenal tumor patients; carbamyl-lysine (CBL).

Biomarker	β1: Age			β2: Sex			β3: Tumor size			β4: Cortisol			β5: Metanephrine			β6: Normetanephrine			β7: Aldosterone		
	Estimate	95%CI	p-value	Estimate	95%CI	p-value	Estimate	95%CI	p-value	Estimate	95%CI	p-value	Estimate	95%CI	p-value	Estimate	95%CI	p-value	Estimate	95%CI	p-value
**CBL**	002,892	−0.08475 to 0.1426	06,121	−1,169	−3.394 to 1.057	02,972	1,423	0.7027 to 2.143	00,002	01,028	−0.08592 to 0.2915	02,797	0,001,108	−0.003678 to 0.005894	06,446	−0,001,952	−0.009124 to 0.005220	05,877	−0,004,709	−0.1153 to 0.1058	09,323

**FIGURE 7:**
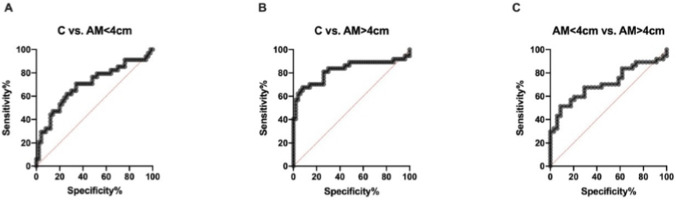
Area under the curve (AUC) of carbamyl-lysine (CBL) plasma content of the controls (C) *versus* patients with adrenal masses <4 cm in diameter (AM<4 cm) **(A)**; the controls *versus* patients with adrenal masses >4 cm in diameter (AM>4 cm) **(B)**; AM<4 cm *versus* AM>4 cm **(C)**.

We checked whether the markers of protein carbamylation differentiate patients with adrenal masses depending on the size of the tumor. We demonstrated possible diagnostic utility only for the plasma content of CBL. This parameter, with moderate sensitivity and specificity, differentiates patients with adrenal tumors: AM < 4 cm (70.59%, 66%) and AM > 4 cm (75.68%, 75%) from the controls (Figure 7; Table 3). Moreover, plasma content of CBL has a diagnostic value in differentiating AM < 4 cm from those AM > 4 cm with sensitivity (67.57%) and specificity (70,59%) (Figure 7; Table 3).

**TABLE 3:** Area under the curve (AUC) of carbamyl-lysine (CBL) of the controls *versus* patients with adrenal masses <4 cm in diameter (AM < 4 cm), as well as patients with adrenal masses >4 cm in diameter (AM > 4 cm); AM < 4 cm and AM > 4 cm patients.

Controls vs. AM < 4 cm								
	AUC	95% CI	p-value	Cut off	Sensitivity %	95% CI	Specificity %	95% CI
CBL	0.8227	0.7208 to 0.9246	<0.0001	>10.53	75.68	59.88%–86.64%	74	60.45%–84.13%

Controls vs. AM > 4 cm								
	AUC	95% CI	p-value	Cut off	Sensitivity %	95% CI	Specificity %	95% CI
CBL	0.6988	0.5796 to 0.8181	0.0021	>9.865	70.59	53.83%–83.17%	66	52.15%–77.56%

AM > 4 cm vs. AM < 4 cm								
	AUC	95% CI	p-value	Cut off	Sensitivity %	95% CI	Specificity %	95% CI
CBL	0.7091	0.5864 to 0.8317	0.0025	>12.47	67.57	51.46%–80.37%	70.59	53.83%–83.17%

## Discussion

Adrenal masses are the most common of all tumors in the adult population (Nieman, 2010). Unfortunately, knowledge about the pathogenesis of adrenal tumors is still limited. This is the first study to evaluate protein glycooxidation, protein oxidative damage, and thiol-disulfide homeostasis depending on the size of the tumor in patients with adrenal masses. We have demonstrated that protein oxidative damage (increase in IMA) and glycooxidation rate (increase in KYN, DT, Amadori products, AGE, and CBL) were significantly higher in patients with adrenal masses than in the control group. Additionally, thiol-disulfide homeostasis (decrease in TT and NT, as well as an increase in DS) was disturbed in these patients. We have also observed that CBL may be a potential biomarker for distinguishing healthy individuals from patients with adrenal masses. Moreover, this parameter was able to differentiate patients with adrenal masses <4 cm in diameter from patients with adrenal masses >4 cm in diameter with moderate specificity (68%) and sensitivity (71%).

TRY is one of the essential amino acids provided in the diet. Unfortunately, in our study, we found reduced TRY plasma content, regardless of tumor size in patients with adrenal masses compared with healthy controls. In physiological conditions, 5% of TRY is used for protein and serotonin synthesis, while 95% is metabolized by indoleamine 2,3-dioxygenase (IDO1, IDO2) and tryptophan 2,3-dioxygenase (TDO2) to NFKYN and then converted by arylformamidase (AFMID) to KYN in the KYN pathway (Bender, 1983; Jones et al., 2013; Perez-Castro et al., 2021). The production and utilization of TRY metabolites, as well as TRY-metabolizing enzyme expression, have been shown to vary between different types of tissues and may be involved in carcinogenesis (Perez-Castro et al., 2021). The decreased levels of TRY were described in colorectal, lung, breast, and prostate cancer (Miyagi et al., 2011; Zińczuk et al., 2021). In our study, we also found decreased content of plasma TRY. Although we have not assessed the activity of enzymes in the KYN pathway, there are reports of increased activity of enzymes involved in TRY metabolism. Indeed, IDO1, TDO2, and AFMID are upregulated in tumors of various origins. Moreover, elevated IDO1 expression correlated with a poor prognosis (Wang et al., 2020; Pallotta et al., 2021). Furthermore, it has been shown that the oncogenic transcription factor MYC increased levels of AFMID and KYN, as well as the TRY transporters SLC1A5 and SLC7A5 in cancer cells (Venkateswaran et al., 2019; Perez-Castro et al., 2021). Accelerated catabolism of tryptophan in neoplasms may lead to excessive accumulation of its metabolites (NFKYN and KYN) (Onesti et al., 2019). KYN is a biologically active compound involved in cell proliferation, promoting and sustaining tumor growth (Perez-Castro et al., 2021; Zińczuk et al., 2021). KYN may act as a specific ligand for the transcription factor AHR, and thus is involved in the regulation of genes that stimulate the proliferation of cancer cells (Venkateswaran and Conacci-Sorrell, 2020; Perez-Castro et al., 2021). The results obtained by us may provide confirmation of this information. Although we did not observe a correlation of plasma KYN content with tumor size, the content of plasma KYN was higher only in patients with adrenal masses >4 cm in diameter, whereas in patients with tumor size <4 cm, its level did not differ significantly from that of the control group. Moreover, KYN may also disturb the function of some cancer-associated immune cells. KYN as well as IDO1 inhibit CD8^+^ T cells, leading to an ineffective anti-tumor response (Frumento et al., 2002; Jiang et al., 2015). The adrenal glands are characterized by a high expression of quinolinate phosphoribosyl transferase, an enzyme involved in tryptophan metabolism in the KYN pathway and *de novo* nicotinamide adenine dinucleotide (NAD) biosynthesis (Foster and Moat, 1980; Youn et al., 2016; Perez-Castro et al., 2021). NAD not only plays a crucial role in redox reactions in the cellular respiratory chain, but also takes part in many cellular processes, genomic stability, aging, and apoptosis (Ying, 2007; Youn et al., 2016). NAD^+^ is also involved in the generation of NADP/NADPH, which protects cells against the harmful effects of oxidative stress. However, in tumor cells, an increased ratio of NAD^+^/NADH levels is observed in comparison to healthy cells, which leads to an intensification of the proliferation and growth process of neoplastic cells (Moreira et al., 2016).

Interestingly, along with the decreased plasma TRY content, we noted an increase in protein glycooxidation products, AGEs, DT, and Amadori products. AGEs are a biomarker of protein carbonyl stress. Their excessive formation occurs through autooxidation of carbohydrates and other glycation mediators (Amadori products) in hyperglycemic conditions (Goldin et al., 2006). By binding to receptors on the cell surface, AGEs can interfere with many intracellular processes. Their connection with a specific receptor, RAGE, is essential. The RAGE receptor has a significant role in carcinogenesis, gene mutations, disturbances in carcinogenesis-related pathways, such as p21/MEK/MAPK, PI3K/AKT/NF-κB, RAS/ERK/p53, and JAK/STAT, as well as angiogenesis, cancer proliferation and metastasis (Bronowicka-Szydełko et al., 2021). CML has been shown to be the main antigenic structure of AGE. CML is recognized by the RAGE for AGE receptor, and the CML-RAGE interaction can activate the NF-κB cell signaling pathway. The AGE-RAGE complex, through the induction of NADPH oxidase system also increases ROS production and activates the NF-κB pathway (Ott et al., 2014). In cancer cells, the excessive accumulation of Amadori products indirectly leads to an increase in the activity of NADPH oxidase and NF-κB expression (Ott et al., 2014; Supruniuk et al., 2020). The consequence of the NF-κB activation is an increase in the expression of cytokines, chemokines, adhesion molecules, growth factors, and inducible nitric oxide synthase (iNOS), as well as enhanced vascular permeability and initiation of inflammatory processes, which take part in tumor pathogenesis through promoting genetic instability, cell growth, and survival (Ahmad et al., 2018). Moreover, disorders of hormone secretion caused by their glycation lead to disturbances of signaling pathways, immune response, and intensification of oxidative stress (Ravichandran et al., 2019). Besides TRY, we also found abnormalities in the content of DT and CBL. Carbamylation is a post-translational modification of proteins as a result of the binding of isocyanic acid from high urea concentrations, leading to the formation of CBL. Carbamylation of proteins causes a partial or complete loss of their function. Under oxidative stress, the ability of proteasomes to remove damaged proteins is limited, which leads to their accumulation inside the cell (Höhn and Grune, 2014). This process, by changing the functions of proteins involved in cellular metabolism, increases the risk of carcinogenesis (Bronowicka-Szydełko et al., 2021). This can be confirmed by the results of our study, in which we observed a relationship between the plasma CBL content and the size of the tumor in patients with adrenal masses. It is not known why carbamylation increases with tumor size, while oxidation and glycation do not. This may be due to the fact that inflammation is the main factor intensifying carbamylation. In our previous work, we observed an increase in serum myeloperoxidase and plasma interleukin-1β in patients with various types of adrenal tumors (Choromańska et al., 2021b). Prolonged activation of MPO may result in increased production of isocyanic acid, which intensifies the process of protein carbamylation (Kisic et al., 2016). However, excessive cytokine production leads to the transformation of healthy cells into cancerous ones (Karin, 2009). It is possible that inflammation, rather than oxidative stress, plays a greater role in carcinogenesis. Furthermore, the production of PC is an effect of the deleterious effects of oxidative and nitrosative stress on amino acids. In the presence of Fe^2+^ and Cu^2+^ ions, ROS and RNS attack the amino acid side chains of arginine, histidine, lysine, proline, cysteine, threonine, and other amino acids (Suzuki et al., 2010). PCs are irreversible products of protein oxidation. In this study, however, we did not find a greater content of plasma PC in patients with adrenal tumors, which has been shown in breast, prostate, and colon cancers (Goswami et al., 2007; Rossner Jr et al., 2007; Zińczuk et al., 2021). In this study, we observed an increase in the plasma content of other carbonyl stress products, IMA. IMA is a product generated from albumin as a result of the action of superoxide radicals’ action under hypoxic conditions (Sbarouni et al., 2011; Stachowicz-Stencel et al., 2011). This parameter is a marker of myocardial ischemia, but its higher concentrations were also found in cancer (Fidan et al., 2012; Zińczuk et al., 2021). Interestingly, Fidan et al. (Fidan et al., 2012) suggested that IMA may be a differentiating marker of gastric cancer.

The human body has developed defense mechanisms against the harmful effects of oxidative stress in the form of thiols. Thiols are organic compounds that contain sulfhydryl groups that have a strong antioxidant effect (Erlic and Beuschlein, 2019; Bin Rubaia’an et al., 2021). Thiols undergo an oxidation reaction to form disulfide bonds that can be reduced back to thiol groups, which maintain the proper functioning of the thiol-disulfide homeostasis. In this study, we found dysregulated thiol-disulfide homeostasis (decrease in TT and NT, as well as increased DS) in patients with adrenal tumors. This is not surprising, since recent research shows that disturbances in thiol-disulfide homeostasis are involved in the pathogenesis of many diseases. Thiols, surprisingly, also have some toxic properties, related to the excessive production of disulfides. Thiol compounds, through the reduction of Fe^+3^ to Fe^+2^, can also show a prooxidative effect through the formation of the thiol radicals and excessive generation of the superoxide radical. This can lead to disturbances in cellular signal mechanisms, regulation of enzymatic activity, transcription factors, and apoptosis (Erlic and Beuschlein, 2019; Bin Rubaia’an et al., 2021).

Our study has several limitations. First, the number of patients was relatively small. Second, the pathology of adrenal tumors and their hormonal activity profiles were heterogeneous, which may explain why most oxidative markers failed to correlate with tumor size. Third, the lack of pathological malignancy scores for most patients included in this study made it impossible to validate the value of CBL as a predictor of malignant risk. In addition, significant differences were observed between the control and patient groups with respect to body mass index, fasting glucose levels, and platelet counts. Since these metabolic and hematologic differences may influence advanced glycation end products and oxidative stress pathways, the observed differences in AGE and glycooxidation parameters may, at least in part, reflect underlying metabolic variability rather than tumor-related processes.

In conclusion, we showed that protein glycoxidation and thiol-disulfide homeostasis are impaired in patients with adrenal tumors. Although oxidative stress is involved in carcinogenesis, in our study, oxidation and glycoxidation of plasma proteins, as well as thiol-disulfide homeostasis, were not dependent on tumor size. Only CBL differentiated patients with adrenal masses <4 cm in diameter from patients with adrenal masses >4 cm in diameter. Based on these preliminary findings, evaluation of plasma CBL content may help predict the size of adrenal masses. More research is needed to confirm our results, not only in plasma but also in tissue, depending on the type of adrenal tumor.

## Data Availability

The raw data supporting the conclusions of this article will be made available by the authors, without undue reservation.
